# The influence of riboflavin and nicotinic acid on *Shigella sonnei* colony conversion

**Published:** 2011-03

**Authors:** AA Deldar, B Yakhchali

**Affiliations:** National Institute of Genetic Engineering and Biotechnology (NIGEB), Shahrak-e Pajoohesh, km 15, Tehran- Karaj Highway, Tehran, Iran

**Keywords:** *Shigella sonnei*, Colony couversion, Riboflavin, Nicotinic acid

## Abstract

**Background and Objectives:**

*Shigella*, causative of bacillary dysentery, has two colony forms. The loss of large virulence plasmid from virulent *Shigella sonnei* form I, during cell storage and subculturing, lead to avirulent form II. Environmental factors, e.g. culture media composition, could affect the conversion of the bacterial forms.

**Materials and Methods:**

In this study, some components, i.e., B-complex vitamins, nicotinic acid and riboflavin, were added to the bacterial culture medium and their influence on colony conversion were examined.

**Results:**

The findings revealed that colony conversion is temperature independent and growth on the SS agar did not stabilize the bacterium in form I. Also, the findings showed that colonies on the minimal media supplemented with nicotinic acid and riboflavin, were stable in form I. In addition, according to the findings, the active OxyR has potential binding sites upstream of two genes involved in the replication of large virulence plasmid and expression of O-polysaccharide, i.e., *repB* and *wbgT*, respectively.

**Conclusion:**

Based on the findings of the present study, it is possible that nicotinic acid and riboflavin activate the transcriptional regulatory protein OxyR via dropping off the intracellular reducing power and in this way stabilize the colonies in form I.

## INTRODUCTION

*Shigella*, causative of bacillary dysentery, i.e., Shigellosis, is a Gram negative bacterium belonging to the *Enterobacteriacae* family. *S. flexneri*, *S. sonnei*, *S. dysenteriae* and *S. boydii are* four subgroups of *Shigella*. The subgroups are divided into several serotypes that are responsible for the infection. Virulent *S. sonnei* has a single serotype determined by O-polysaccharide of form I. *S. sonnei* is responsible for the endemic form of the disease in the most areas of the world (1, 2).

Although a variety of candidate vaccines, including recombinant heterologous live bacterial carrier strains, have been developed (2), no efficient vaccine against the bacterium is available (3). *S. sonnei* form I is unstable and through the spontaneous loss of the large virulence plasmid converts to an avirulent form. While provoking a strong immune response entails an invasive (penetrating) vaccine strain (form I), this avirulant form cannot penetrate into the epithelial cells to induce immune response (4–6). Since during cell storage and subculturing, the large virulence plasmid tends to be lost at a high frequency, the attenuated vaccine strains of the bacterium may convert to non-penetrating form and consequently could not be presented to the immune system through dendritic cells. This phenomenon is limiting in vaccine development programs. Therefore, stabilization of the virulence plasmid may provide a condition in which an attenuated *S. sonnei* strain maintains its penetrating properties which are necessary for efficiency of the vaccine strain. Thus, further studies are needed to stabilize the *S. sonnei* form I (2, 7, 8).

To stabilize the bacterium in the penetrating form, the differences between form I and form II of *S. sonnei* should be inspected. The conversion of form I to form II is detectable on the nutrient agar plate. Form I colonies are round and raised with a regular margin, whereas form II colonies appear flat and rough with an irregular margin (4).

Virulent *S. sonnei* form I synthesizes a complete LPS structure, which is a clearly defined bacterial virulence factor necessary for penetrating epithelial cells. A cluster of nine essential genes encoding O-antigen biosynthesis, located on the pSs046 plasmid. The loss of this gene cluster, like the missing of the large virulence plasmid, results in form II colonies that are not able to synthesize O-specific antigen (2, 4, 9, 10).

Since the critical difference between forms I and form II colonies is the presence of O-antigen side chains in form I and their absence in form II (11), changes in morphotype may be due to alteration in the transcription of O-antigen coding genes and/ or genes involved in the maintenance of the large virulence plasmid.

Like other bacterial processes, maintenance of the virulence plasmid and expression of O-antigen may be under regulatory control. One potent regulatory gene that may have a role in these events is *oxyR*. OxyR is a peroxide-sensing, transcriptional regulatory protein. The oxidative stress signal is transduced by oxidation of two cysteine residues in OxyR, which leads to the formation of an intramolecular disulfide bridge. This event accompanied by changes in protein conformation and protein-DNA interaction (12, 13). Deletion of the *oxyR* in *E. coli* has confirmed its contribution in the control of some surface properties, including colony morphology (14).

Probably, *oxyR* exerts its control on the form I appearance through control of the O-polysaccharide biosynthesis gene cluster or gene(s) involved in the large virulence plasmid replication, i.e., *repA* and its repressor *repB* (15, 16).

Given that during oxidative stress a decrease in the NADPH/NADP^+^ ratio and an increase in the FAD/ NAD(P)H ratio redox indicators can be observed (17), the purpose of the present research was to alter the redox indices and by doing so change the intracellular reducing power and consequently activate the OxyR. Therefore, components such as B-complex vitamins, nicotinic acid and riboflavin that their derivatives play crucial roles in the cellular redox reactions- were added to the bacterial culture media and their effect on the colony conversion were examined.

## MATERIALS AND METHODS

**Plasmid, bacterial strains and culture media**. Recombination plasmid pKOBEG (provided by Patricia Latour Lambert; Pasteur Institute Paris) were used for gene replacement.

Strains of wild type *S. sonnei* (provided by Bahar medical diagnostic laboratory, Tehran, Iran) and mutant *S. sonnei*Δ*ipab* <*bla*>, constructed using a recombineering plasmid pKOBEG, were used in this research.

Culture media were Tripticase Soy Agar (TSA), Salmonella Shigella (SS) Agar, Luria-Bertani broth (LB) and Luria-Bertani agar (LA), LA supplemented with glycerol as carbon source (LAG), Terrific Broth (TB) and Terrific agar (TA) containing glycerol, TA- amp (200 µg/ml) and M9 basic medium containing glycerol and B-complex vitamins (M9GB; composed of thiamine Hydrocloride 5 µg/ml, Riboflavin 2 µg/ml, Pyridoxine hydrochloride 2 µg/ml, Nicotinamide 20 µg/ml and Dexpanthenol 3 µg/ml), M9 containing glycerol and riboflavin 25 µg/ml (M9GR), M9 containing glycerol and 25 µg/ml nicotinic acid (M9GN), M9 containing 25 µg/ml riboflavin and 25 µg/ml nicotinic acid (M9GRN) and M9 containing glycerol and 1% casamino-acids (M9GCA). Final concentration of glycerol in all culture media was 0.4% v/v.

**Colony morphotype analysis**. For colony conversion experiments, *S. sonnei* strain isolated from patient were streaked on TSA and incubated at 37°C overnight. In order to observe colony conversion, one smooth colony was selected and resuspended in sterile double distilled water. 100 µl of the bacterial suspension containing 103 cells /ml, was spread on each of the SS agar, LA, TA media with and without B-complex vitamins, LAG, M9GB, M9GR, M9GN, M9GRN and M9GCA media.

To analyze the effect of the two growth temperatures i.e., 30 and 37°C, on the colony form, one smooth colony from overnight culture of Terrific agar at 30°C was selected and resuspended in sterile double distilled water and 100 µl of the bacterial suspension containing 103 cells /ml, was spread on each of the M9GB, TA and LA and incubated at 30 and 37°C for 24 hours. The colonies with round margins were considered as form I and the colonies with irregular margin as form II.

**Polymerase Chain Reaction**. Genomic DNA of *S. sonnei*, *E.coli*, and *Pseudomonas fluorescence* were used to amplify 16s rDNA. Smooth colonies from M9GB and TA, and rough colonies from TA were used for chromosomal DNA preparation (18). Genomic DNA of smooth and rough colonies were used for the amplification of the *ipaB* gene. Descriptions and the sequences of the PCR primers are given in Table 1.

**Table 1:** PCR primers used to amplify specific DNA fragments.

Primer	Description	Sequence	Amplicon size (bp)
*En16-1*	Forward primer for *16s* rDNA	5'-AGA CAA TCT GTG TGA GCA C-3'	1646
*En16-2*	Backward primer for *16s* rDNA	5'-CCA AGT CTC AAG AGT GAA C-3'	
*Ss-1*	Forward primer for *rrsH*	5’-AAC TTT GCA GAG ATG GAT TG-3’	713
*Ss-2*	Backward primer for *rrsH*	5'-AAG ACC TCT TCA AAT TTG CG-3'	
*Ss.ip-1*	Forward primer for *ipaB*	5'-ATC ATA CTT GGA CGC AAT TCA GGA TAT CAA GGA GTA ATT ATT ATG-3'	1831
*Ss.ip-2*	Backward primer for *ipaB*	5'-GGT TGA TTT TGT GTT TTG AAT TTC CAT AAC ATT CTC CTT ATT TGT ATC-3'	

Enzymes and chemicals were provided by Cinnagen Chemical Company (Tehran, Iran). Amplifications were performed in a thermal cycler (Techgen, Germany). PCR was performed in a 25 µl reaction containing 50 mM KCl, 10 mM Tris–HCl (pH 8.3), 1.5 mM MgCl, 1.l%gelatin, 0.2 mM dNTPs, 1.25 units Taq or pfu DNA polymerase, and 10 pmol each of the forward and reverse primers.

The thermocycling parameters used in PCR were as follows: 2 min of initial denaturation at 94°C and 30 cycles of denaturation, 20s at 94°C; annealing, 30s at either 50°C (*S. sonnei* rRNA; *rrsH* and*16s rDNA*) or 65°C (*ipaB* coding gene); extension, 2 min at 72°C. Final extension was done 5 min at 72°C.

**Bioinformatics**. Sequences of *repB* and *wbgT* genes and their upstream sequences were downloaded from *Gene Bank* (accession No. CP000038) and analyzed by “Virtual Footprint Online Software” for the presence of OxyR binding site (19) and by online software “BPROM Prediction of bacterial promoters of Softberry” for promoter prediction. All primers were analyzed by *in silico* simulation of PCR (20).

Sequencing was performed with DyeDeoxy Terminator cycle sequencing kits (Applied Biosystems) and an ABI model 373A automated sequencer (MWG, Germany).

## RESULTS

**Fig. 1:**
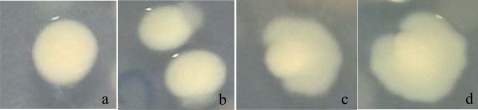
Conversion of the smooth colony of *S. sonnei* to a rough colony on TA and/or LA medium, a: a colony in form I, b: beginning of colony conversion, c: a semi-converted colony, d: a colony before complete converting to form II.

In general, form II phenotype was diverged from the growing *S. sonnei* form I on TA and LA media. Some of them were started converting at once and the resulting colonies were not appeared as form I at all. But other colonies primarily were emerged in form I and then started to convert (in a budlike manner). After 72h, all of the smooth colonies were completely surrounded by a rough region with irregular margin (Fig. 1).

**Fig. 2:**
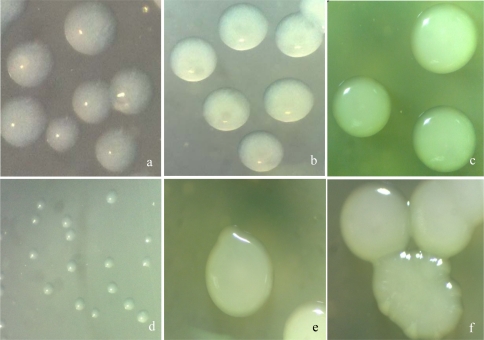
Appearance of S. sonnei colonies after 48h on M9GB (a), M9GN (b), M9GRN (c), M9GR (d); Bud-like shape on M9GRN (e); the form II of the bacterium on the M9GRN (f). Colonies on M9GR(d) were smaller than 0.3 mm (Ma =7×)

On the M9GB, TA and LA media after 72 hours incubation at 30 and 37°C, just the colonies on the M9GB remained in form I (Table 2). *S. sonnei* colonies on the M9GB, M9GN and M9GRN media were small, smooth and round (form I). Approximately, 0.2% of the colonies on the M9GRN appeared in form II (Fig. 2f). While, both colony types appeared on the TA and LA were large, the ones on M9GCA were small, (Table 3). After 48 hours, the size of colonies on the M9GB, M9GN, M9GRN, and M9GCA media varied from 1–2, 0.7–1.5, 1–2 and 1–2 millimeter respectively. Prolonged incubation of the plates until 72h did not alter the appearance of colonies on the M9GB, M9GN and M9GRN media.

**Table 2:** Colony morphotypes of *S. sonnei* on different culture media at 30 and 37°C.

Media	M9GB	TA	lA
Tm			
30°C	I	I/II	I/II	
37°C	I	I/II	I/II	

*Note: I=Form I; II=Form II

**Table 3:** Colony morphotypes of *S. sonnei* on different culture media after 48h.

	TA	TAB	LA	LAB	LAG	M9GB	M9GR	M9GN	M9GRN	M9GCA	SS agar
Colony form	I/II	I/II	I/II	I/II	I/II	I	NG	I	I	I/II	I/II
Form I percentage	87.4	91.2	87.9	91	90	100	--	98.8	100	94.8	93
Colony size (mm)	≥4		≥4		≥4	1–2	<0.5	0.7–1.5	1–2	1–2	≥4

*Note: I=Form I; II=Form II; NG=bacteria did not have noticeable growth; mm=millimeter.

**Fig. 3:**
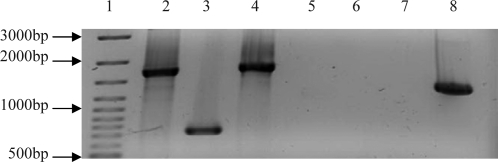
Electrophoresis of PCR products of 16s rDNA from *S. sonnei*, *E. coli DH5-α* and *P. fluorescence*. Lane1: DNA marker 100 bp (Fermentas); Lane2: *S. sonnei 16s rDNA*,1646 bp fragment; Lane3: *S. sonnei* specific rrsh,714 bp fragment; Lane4: *E.coli 16s rDNA*,1646 bp fragment; Lane 5: *E. coli* DNA amplification by *Ss.1 and Ss. 2* Primers; Lanes 6&7: *P. fluorescence* DNA amplification by *En*., and *Ss*. primers respectively; Lane 8: PCR Control (*flp*)

The verification of the two morphotypes of *S. sonnei* by conventional biochemical tests revealed that except maltose fermentation by form I colonies, all of their properties were the same (data did not shown). When *S. sonnei* genomic DNA were used for amplification of *16s* rDNA and *rrsh* rDNA, both of the fragments were amplified. While due to the species specificity of *rrsh* primers, only *16S* (1646 bp) rDNA fragments were amplified for *E.coli* genomic DNA, none of the fragments were amplified for *P. fluorescence* (Fig. 3).

**Fig. 4:**
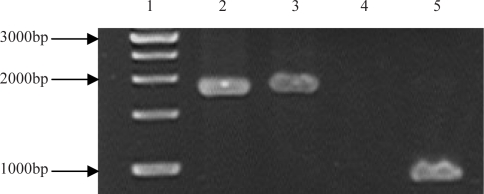
The amplified *ipaB* gene of *S. sonnei* morphotypes. Lane 1: DNA marker 1 kb (Fermentas); Lane 2: Smooth colony from TA; Lane 3: Smooth colony from M9GB; Lane 4: rough colony from TA; Lane 5: PCR Control (*flp*).

Amplification of *ipaB* gene was used to determine the presence of the large virulence plasmid in the bacterium as a molecular marker of *S. sonnei* form I. As it was expected, DNA fragment of 1831bp of *ipaB* gene was amplified from total DNA of form I colonies. The fragment was not amplified from total DNA of form II colonies (Fig. 4).

**Fig. 5:**
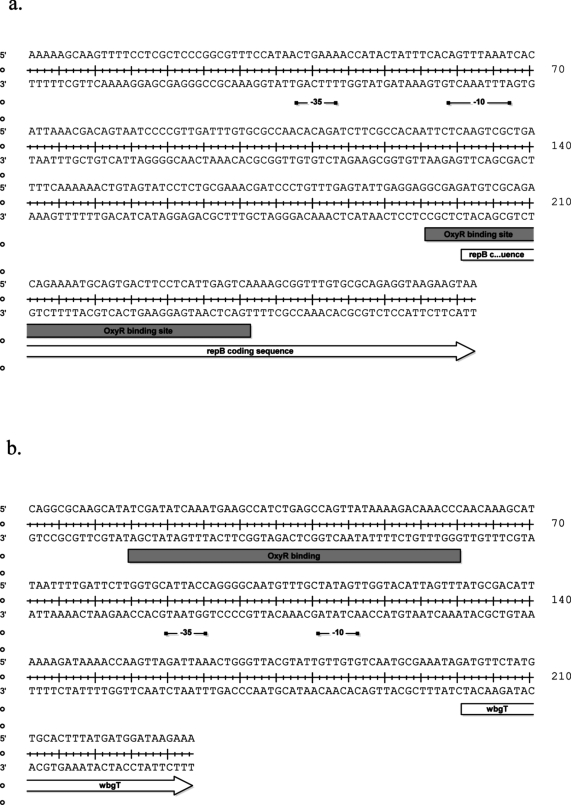
Sequences of *repB* (A) and *wbgT* (B) genes and their upstream regions Gray box indicate the potentially OxyR binding sites. White box indicates promoter and arrow indicates coding sequence (partial).

As probable target genes for oxidized active OxyR, the *repA*, *repB* and *wbgT* (the first gene of O-polysaccharide biosynthesis gene cluster) and their upstream regions were analyzed. The potential binding sites for this regulatory protein were extended from 123 to 169 of *repB* (Fig. 5a) and from −110 to −65 of *wbgT* relative to transcription initiation sequence (Fig. 5b). The *repA* did not have any potential binding site for the OxyR.

Briefly, *S. sonnei* on the minimal media supplemented with B-complex vitamins or nicotinic acid and riboflavin were grown in form I. The colonies on these media were maintained large virulence plasmid. The active OxyR, formed in the presence of oxidizing conditions, has potential binding sites at the upstream of two genes involve in the replication of large virulence plasmid and expression of O-polysaccharide i.e., *repB* and *wbgT*, respectively.

## DISCUSSION

The findings of the present study, in consistence with the findings of Farshad *et al*. (8) and Warne *et a*l. (14), revealed that *S. sonnei* colonies on the TA and LA media convert to form II. This is possibly due to the fact that the large plasmids tend to be lost during cell storage and subculturing. When a form I colony of *S. sonnei* was suspended and spread on enriched agar plates, after 24 hours incubation, about 10% of the resulting colonies were switched to form II (5). In contrast with the findings of Sayeed et al. (26), this colony conversion was temperature independent and the growth in 30°C did not prevent the colony switching. Also, contrary to Branham et al. (21), SS agar did not stabilize the bacterium in form I. The colonies on the M9GB, M9GN and M9GRN grew up as smooth colonies and prolonged incubation of these plates did not alter the appearance of the colonies on the media. Although 0.2% of the colonies on the M9GRN developed in form II, the rate of conversion on this medium did not significantly differ from the conversion rate on M9GB and M9GN (p≥95).

In fact, the colonies on M9GB and M9GRN have a larger size than the ones on M9GN. Therefore, M9GRN that contains riboflavin and nicotinic acid, could be used for the growth of *S. sonnei* form I. In other words, riboflavin and nicotinic acid can play a crucial role in the growth of *S. sonnei* form I. We believe that the presence of riboflavin and nicotinic acid in these media can be manifested as an external pressure (such as that which occurs under oxidative stress) which fixes the colonies in form I.

The findings suggest that in the presence of nicotinic acid and riboflavin as an anti-reducing condition on the minimal medium, the rough colonies might not be developed. In other words, the loss of virulence plasmid occurred in the absence of the condition exerted by riboflavin and nicotinic acid.

To clarify the role of nicotinic acid and riboflavin in colony fixing, their *in vivo* functions should be elucidated. Riboflavin acts as a central component of the cofactor FAD. It seems that increasing FAD may lead to an increase in FAD/NAD (P) H ratio -a redox indicator which can also be observed during oxidative stress (17). Therefore, adding riboflavin to the medium may result in a condition similar to the one occurs during oxidative stress.

Nicotinic acid is a crucial component for the noticeable growth of the bacterium. *In vivo*, nicotinic acid is converted to NAD (P). As an electron carrier, NAD(P) is involved in redox reactions of cellular metabolism. Decreasing of NADPH or increase of NADP^+^ lead to shrinking of the NADPH/NADP^+^ ratio that in turn, drop off intracellular reducing power (22).

In the absence of oxidative stress the cytoplasm is a reducing environment and the main form of the OxyR is reduced. Dropping off the intracellular reducing power lead to alterations in the cellular redox state that changes the redox status of some regulatory proteins including OxyR. Like oxidative stress, changing of the redox status as a signal is transduced by the activated OxyR. By this way, transcriptional pattern of the bacterium may affect and the properties of bacterium including morphological ones may be changed (13, 22, 23).

Deletion of the *oxyR* gene from a strain of *E. coli*, which exhibits switching between Forms I and form II, have fixed the strain in the rough form permanently (14). Since the critical difference between Forms I and form II colonies is the presence of O-antigen side chains in form I and their absence in form II (11), changes in the morphotypes may be due to alteration in the transcription of O-antigen coding genes and/or genes involved in the maintaining of virulence large plasmid.

As a transcriptional activator, the activated OxyR participates in the recruitment of RNA polymerase to the target genes (24). Several genes, including *rcsC* (encoding a sensor-regulator protein of capsular polysaccharide synthesis genes) (25), *repB*, *wbgT* (19), and *fhuF* interact with OxyR for expression or repression.

Because RepB represses the *repA* gene transcription and OxyR has a potential binding site on the initiation sequence of *repB* gene, it seems that the nicotinic acid and riboflavin in minimal medium decreases the intracellular reducing power and activates reduced OxyR, which may repress *repB* transcription. Therefore, the replication of the virulence plasmid initiates by RepA and segregation of the replicated plasmids during bacterial proliferation results in form I progenies.

Xu et al. (2002) proposed that nine genes beginning with *wbgT* and ending with *wbgZ* are required for biosynthesis of O-polysaccharide in *S. sonnei* form I (2). The *wbgT* gene, which encodes putative UDP-glucose 6-dehydrogenase, has a potentially OxyR binding sequence from −110 to −65 relative to transcription initiation sequence (Fig. 5b) (2, 19). Therefore, it seems that this gene is under the OxyR positive control and can express when the intracellular reducing power decreases.

Apparently, in the defined media (M9GB and M9GRN), the bacteria take up riboflavin and nicotinic acid from the culture media which results in the intracellular reducing power decline. Under such circumstances, the OxyR may tend to be oxidized. The oxidation activates this transcriptional regulatory protein which may lead to the binding to the related target sites at the upstream of several genes. Inducing or inhibiting of the transcription of genes such as *wbgT* and *repB* may be the subsequent outcomes of the process. Turning off the *repB* may prevent the loss of virulence plasmid, while turning on the O-polysaccharide biosynthesis gene cluster can lead to the synthesis of O-antigen side chain of LPS. In fact, the repression of the *repB* and expression of the O-antigen coding gene cluster in the presence of riboflavin and nicotinic acid possibly result in the fixing of the colonies in form I.
